# Assessment of the Risk Impact of SARS-CoV-2 Infection Prevalence between Cats and Dogs in America and Europe: A Systematic Review and Meta-Analysis

**DOI:** 10.3390/pathogens13040314

**Published:** 2024-04-12

**Authors:** Marcos Jessé Abrahão Silva, Davi Silva Santana, Marceli Batista Martins Lima, Caroliny Soares Silva, Letícia Gomes de Oliveira, Ellerson Oliveira Loureiro Monteiro, Rafael dos Santos Dias, Bruna de Kássia Barbosa Pereira, Paula Andresa da Silva Nery, Márcio André Silva Ferreira, Matheus Alonso de Souza Sarmento, Andrea Alexandra Narro Ayin, Ana Cristina Mendes de Oliveira, Karla Valéria Batista Lima, Luana Nepomuceno Gondim Costa Lima

**Affiliations:** 1 Center for Biological and Health Sciences (CCBS), University of the State of Pará (UEPA), Belém 66087-670, PA, Brazil; karolinysoares2303@gmail.com; 2 Institute of Health Sciences (ICS), Institute of Biological Sciences (ICB), Federal University of Pará (UFPA), Belém 66077-830, PA, Brazil; doc2022davi@gmail.com (D.S.S.); marceliblima@gmail.com (M.B.M.L.); rafa.santo.dias@gmail.com (R.d.S.D.); masfmedufpa@gmail.com (M.A.S.F.); cris.mastozoologia@gmail.com (A.C.M.d.O.); 3 Evandro Chagas Institute (IEC), Ananindeua 67030-000, PA, Brazil; gomes_15_letici@hotmail.com (L.G.d.O.); karlalima@iec.gov.br (K.V.B.L.); luanalima@iec.gov.br (L.N.G.C.L.); 4 Instituto Federal do Pará (IFPA), Belém 66645-240, PA, Brazil; ellerson987@gmail.com; 5 Department of Veterinary Medicine, University of the Amazon (UNAMA), Belém 66120-901, PA, Brazil; vetbruna1@gmail.com (B.d.K.B.P.); pasnmed@gmail.com (P.A.d.S.N.); 6 Faculty of Medicine, Centro Universitário do Estado do Pará (CESUPA), Belém 66613-903, PA, Brazil; matheusalonsos@hotmail.com (M.A.d.S.S.); andreaayin@hotmail.com (A.A.N.A.)

**Keywords:** SARS-CoV-2, disease etiology, public health, zoonosis, animals

## Abstract

The COVID-19 pandemic represented a huge obstacle for public health and demonstrated weaknesses in surveillance and health promotion systems around the world. Its etiological agent, SARS-CoV-2, of zoonotic origin, has been the target of several studies related to the control and prevention of outbreaks and epidemics of COVID-19 not only for humans but also for animals. Domestic animals, such as dogs and cats, have extensive contact with humans and can acquire the infection both naturally and directly from humans. The objective of this article was to summarize the seroprevalence findings of SARS-CoV-2 in dogs and cats and correlate them with the strength of infection risk between each of them. This is a systematic review and meta-analysis following the recommendations of PRISMA 2020. The search and selection of papers was carried out using in vivo experimental works with animals using the descriptors (MeSH/DeCS) “Animal”, “Public Health”, “SARS-CoV-2” and “Pandemic” (together with AND) in English, Portuguese or Spanish for Science Direct, PUBMED, LILACS and SciELO databases. The ARRIVE checklist was used for methodological evaluation and the Comprehensive Meta-Analysis v2.2 software with the Difference Risk (RD) test to evaluate statistical inferences (with subgroups by continent). Cats showed greater susceptibility to SARS-CoV-2 compared to dogs both in a joint analysis of studies (RD = 0.017; 95% CI = 0.008–0.025; *p* < 0.0001) and in the American subgroup (RD = 0.053; 95% CI = 0.032–0.073; *p* < 0.0001), unlike the lack of significant difference on the European continent (RD = 0.009; 95% CI = −0.001–0.018; *p* = 0.066). Therefore, it was observed that cats have a greater interest in health surveillance due to the set of biological and ecological aspects of these animals, but also that there are a set of factors that can influence the spread and possible spillover events of the virus thanks to the anthropozoonotic context.

## 1. Introduction

Coronavirus disease 2019 (COVID-19) is an infectious disease caused by severe acute respiratory syndrome (SARS-CoV-2), a type of beta-coronavirus that has infected around 3 billion people worldwide, and more than 500,000 deaths have been recorded [1]. The COVID-19 pandemic has revealed hidden social problems in healthcare systems, social equity and environmental management with positive and negative socio-economic effects in the short and long term due to COVID-19 lockdowns in social, environmental and economic environments [2].

With regard to the causality of diseases, it is known that COVID-19 in humans was transmitted zoonotically, and its spread occurred through spillover of the agent between different species [3]. Scientific evidence demonstrates that bats were the ancestral reservoirs of SARS-CoV-2 and were able to concentrate this new type of virulence through a process of genetic recombination between the bats’ own reservoirs [4]. They can also act as reservoirs for several other emerging zoonotic pathogens such as Nipah virus, Hendra virus, influenza virus, Ebola virus, rabies virus and CoVs [5].

Coronaviruses (CoVs) can withstand a range of environmental conditions and infect a wide range of mammal and bird species. CoVs alter or expand their host range, which may contribute to the possibility of creating new CoV variants in humans. This fact was previously evidenced by discoveries of CoV tissue tropism and variations in its host range. Such information is reported from cases such as in animal tissue cultures, where these new variants already exist/emerge as quasi-species of viruses during replication or they spread in other avian/mammal species [6]. CoVs react and evolve rapidly to adapt to different hosts, tissues and environments, even if they are impacted by anthropogenic and perhaps climatic influences [7]. It is assumed in most studies that environmental factors such as temperature, humidity, climate and ventilation have an impact on various aspects of the transmission chain [8].

Natural infection by the virus has been identified in different animal species. These correspond to cats, dogs, minks, cougars, gorillas, lions, snow leopards, tigers, ferrets and otters in more than 25 countries [2]. Furthermore, among wild animals, mainly carnivores, great primates and white-tailed deer have been reported to be naturally infected with SARS-CoV-2 [9]. There is evidence that cats, ferrets and hamsters are among the companion animals most at risk of infection with SARS-CoV-2, which probably originates most often from infected humans themselves, and which has little impact on the circulation of the virus in the human population [10].

Furthermore, SARS-CoV-2 can cause subclinical infection in cats and dogs, although its transmission to humans is considered uncommon [11]. Although uncommon, there was one case already registered in Europe of transmission of a variant of SARS-CoV-2 from minks, which had been infected by COVID-19, to mink workers [12]. In this sense, the risk that wild or farmed animals represent for the transmission of SARS-CoV-2 is also important to be investigated, so that effective recommendations and risk management measures against COVID-19 can be made [13].

Companion animals have very close contact with humans in close environments. As companion animals such as dogs and cats are potential sources and sentinels of a wide range of infections, such as SARS-CoV-2, determining their susceptibility to them and natural SARS-CoV-2 infections has significant impact on animal and human health. Therefore, the infection of dogs and cats occurs mainly through human transmission and not naturally between pets. Despite it being known that cats are more susceptible to SARS-CoV-2 than dogs, there are still gaps in understanding both the possibility of natural infection in these animals and in measuring this risk of infection by the aforementioned animals [14]. In this sense, this work takes a One Health approach (animal–human interaction) to conduct a systematization of the difference in the proportion of risk of SARS-CoV-2 infection between cats and dogs.

## 2. Material and Methods

### 2.1. Study Design

This is a systematic review with meta-analysis using a descriptive and inferential approach related to the specificities of the epidemiological relationship of viral prevalence in dogs and cats to the transmission chain of the SARS-CoV-2 agent. This bibliographic study aims to obtain an overview of this animal–human clinical problem investigated throughout the world and to the detriment of the American and European continents.

### 2.2. Formation of the Guiding Question

To create the research problem, the POT strategy was used, with the acronym standing for the following: patient or problem (P), outcome (O) and type of study (T) [15]. For this, it was characterized into problem (P): dogs and cats exposed to SARS-CoV-2; outcome (O): determine the difference in risk proportion between dogs and cats having SARS-CoV-2; type (T): experimental studies. In this sense, the following problem arises: “What is the estimated difference in risk between dogs and cats having SARS-CoV-2?”.

### 2.3. Search Strategy

The research was carried out in the following databases: Latin American and Caribbean Literature in Health Sciences (LILACS), the National Library of Medicine Institutes of Health of the USA (PubMed), the Scientific Electronic Library Online (SciELO) and Science Direct. The literature search mechanism was defined by the use of advanced search in individual databases combined with the Boolean operator AND, through the following keywords/descriptors (MeSH/DeCS): “SARS-CoV-2”, “Public Health”, “Animals” and “Pandemic”. For better accuracy of the results, the following filters were used: language, time frame and type of study. In this sense, the selection of relevant filter items was English, Spanish or Portuguese studies outlined in the period from December 2019 to December 2023 and texts defined as primary.

### 2.4. Inclusion and Exclusion Criteria

The inclusion criteria were defined as complete available articles relevant to the research question with a One Health theoretical perspective, free access and of the experimental study type. The One Health scientific and health perspective consists of a collaborative and multidisciplinary strategic framework that focuses on reducing the risk and minimizing the global impact of epidemics and pandemics due to emerging infectious diseases through the provision of effective response and control of them, considering a causality and ecological interaction between environmental, animal and human factors for understanding zoonoses [16]. The exclusion criteria were epidemiological articles focusing on humans, those published prior to December 2019, articles that were duplicates, articles where only the abstract was available, articles with inaccessibility to important information in the article and articles with topics not relevant to the research question.

### 2.5. Data Extraction

Data extraction from articles was carried out using Excel software (Microsoft Office 365, v. 15.0) to organize and screen the review results. Data extraction was conducted in tabular form and took into account the following characteristics: author and year of publication, title, methodology, database, classification and biological taxonomy of the respective animals, results and country of origin of the study animals. The data in this review sought to cover studies on the domestic animals in focus (dogs and cats, independently), but they also dealt with primary data on dogs and/or cats in their analyses. Conversely, the statistical and inferential part only took into account articles in which dogs and cats were evaluated in the same investigation by a specific author. This way of conducting the study was used to make its discussion more robust.

### 2.6. Methodological Assessment of Studies and Data Synthesis

Reports of in vivo experiments with animals were methodologically evaluated using the Animal Research: Reporting of In Vivo Experiments (ARRIVE) checklist. In it, a maximum score of 20 points was assigned to each article evaluated independently by 2 authors (MJAS and DSS), under the supervision of a third researcher (MBML). Based on the total score, the studies were categorized into three different groups (good, moderate and bad), in relation to the percentage acquired by the score (if it was ≥75%, ≥65% and ≤55%, respectively).

For data synthesis, the studies were presented according to the following categories: author, year, title, methodology, study population, country and results. The PRISMA flowchart, based on the PRISMA protocol, was used to present the steps followed for the present study [17,18].

### 2.7. Statistical Analysis

Comprehensive Meta-Analyses (CMA) program, version 2.2 (Biostat, Englewood, NJ, USA), was used on a computer to perform the statistical analyses of the meta-analysis. The fixed-effects model was used, using a 95% confidence interval (95% CI). A subgroup analysis was performed to determine whether there was greater weight in relation to the continents evaluated. The Cochrane Chi-Square test and the I-square measure (I^2^) were used to determine the considerable statistical difference (*p* ≤ 0.05 was considered a significant threshold) [19]. The correlation test for Begg and a funnel plot were used to examine the potential for publication bias (*p* ≤ 0.05 was considered statistically significant) [20].

## 3. Results

### 3.1. Characteristics of the Selection of Studies by Database

**Figure 1:**
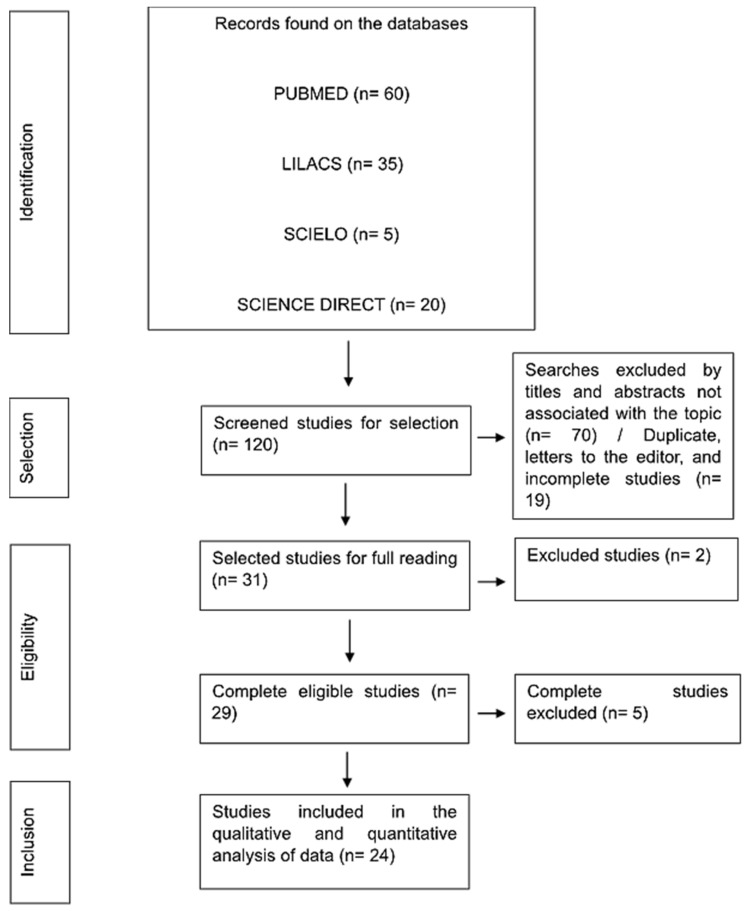
Flowchart of criteria for identification, selection, eligibility and inclusion of data in the meta-analysis. Belém, Pará, Brazil (2024).

To track possible works in the analyzed databases, the initial search through the descriptors in the databases found 120 articles to identify possible selections. Regarding their selection, reading the title and abstract and checking the type of article found made it possible to exclude 89 papers. The complete reading of the articles defined by the eligibility criteria and extraction of relevant data required a sample number of seven surveys. Thus, the final literary composition included was 24 articles (Figure 1).

### 3.2. Characteristics of the Studies Added in This Review

The final sample consisted of 24 articles, most of which came from the USA (*n* = 5; 20.83%) and the American continent (*n* = 9; 37.5%), from the PUBMED database (*n* = 24; 100%), using a laboratory method such as ELISA (*n* = 14; 58.33%), which obtained the data shown in Table 1.

After the methodological quality analysis, it was observed that no study had low/low quality (0%), with the majority being moderate (*n* = 16; 66.67%), while only eight were considered good (33.33%), indicating that the studies were well designed and well conducted for their final inclusion in the meta-analysis. These findings are displayed in Table 2.

### 3.3. Forest Plot and Funnel Chart of the Meta-Analysis Produced

**Figure 2:**
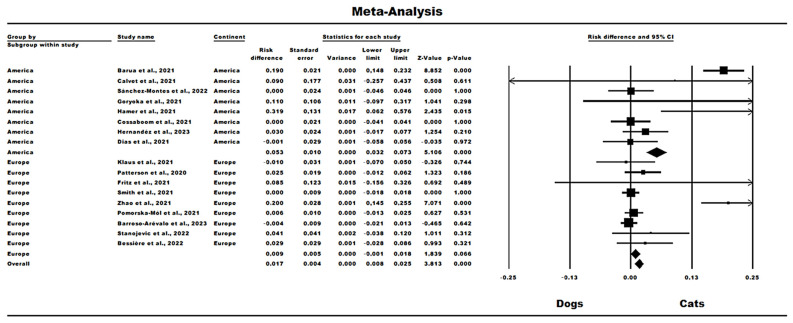
Forest Plot of the Meta-analysis regarding the difference in risk of SARS-CoV-2 infection in dogs and cats in America and Europe [21,22,23,24,25,26,27,28,29,30,31,32,33,34,35,36,37,38,39,40,41,42,43,44].

The meta-analysis consisted of 18 eligible studies (with primary studies that analyzed dogs and cats simultaneously) for inferential investigation of the summarized data. The resulting risk difference (RD) worldwide was significant in relation to the greater risk of cats becoming infected than dogs (pooled difference [RD], 0.017; 95% confidence interval [CI], 0.008 to 0.025, *p* < 0.0001). Regarding the subgroups of continents analyzed, the American continent had significant results in relation to the greater proportional number of cats infected by SARS-CoV-2 in terms of the risk of infection compared to dogs (pooled difference [RD], 0.053; 95% confidence interval [CI], 0.032 to 0.073, *p* < 0.0001), while on the European continent, no significant associations were found for risk differentiation (pooled difference [RD], 0.009; 95% confidence interval [CI], −0.001 to 0.018, *p* = 0.066) (Figure 2).

**Figure 3:**
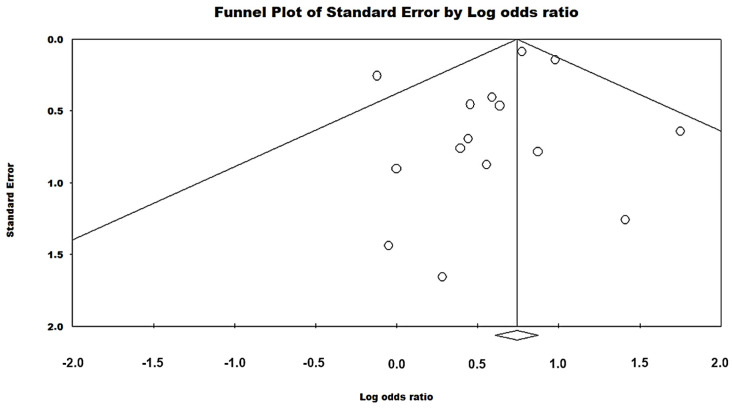
Funnel plot of this meta-analysis. The symmetry of the graph is represented by the proportional and relatively well-distributed division on both sides of the horizontal axis of the circles in its plane [21,22,23,24,25,26,27,28,29,30,31,32,33,34,35,36,37,38,39,40,41,42,43,44].

Regarding the data analyzed on heterogeneity between studies, the meta-analysis overall had a rate of 26.11% (χ^2^ = 18.95; df = 18; *p* = 0.16). With regard to the subgroups evaluated, the American continent showed 0% heterogeneity (χ^2^ = 2.97; df = 5; *p* = 0.70), while Europe had low heterogeneity, that is, 26.11% (χ^2^ = 18.95; df = 14; *p* = 0.05). The funnel plot was considered symmetrical, with no evidence of high publication bias and the Begg test (*p* = 0.62), that is, no statistical significance in the presence of bias (Figure 3).

## 4. Discussion

The concept of One Health, aimed in particular at endemic zoonoses of public health concern, focuses on the main outcomes, causalities and interactions at animal–human–ecosystem interfaces [45]. Fortunately, compared to humans, many domestic and companion animals are less vulnerable to SARS-CoV-2 [46]. The weak sensitivity of animals, such as dogs and cats, compared to humans is due to intrinsic host factors, such as the functioning of the angiotensin-converting enzyme 2 (ACE2) (the main cellular receptor for viral entry into the host) and certain proteases [47].

In the same way as in previous studies, it was demonstrated here that cats may have greater susceptibility to SARS-CoV-2 than canines, especially in the American continent [10,48,49]. The greater susceptibility of cats to the highlighted virus is related to environmental, biological and ecological factors. According to a study by Chen et al. (2020), the percentage of cells containing ACE2 and TMPRSS2 (receptors crucial for viral adsorption) was absent in chickens, was low in pigs, was very rare in dogs but was high in cats [50]. Dogs are a species less susceptible to viral infection, which usually manifests asymptomatically with little viral excretion. This is probably due to the fact that dogs have a lower expression of ACE2 receptors in the respiratory system, which contributes to poor virus replication [51].

One of the other reasons that may be behind the factors that enable the greater infection of cats in relation to other animals is in data on the spatial ecology of these animals (referring to the propagation, persistence and interactions of individuals in landscapes through dispersal), since cats generally have a high rate of movement across locations, which can lead to greater spread and transmission of the virus to them [52]. On the other hand, in other regions of the world, the growing global urbanization and possible lockdowns related to the pandemic may have represented a loss of space and previous environments belonging to cats and dogs, which remained closer to their owners for longer and may be more conducive to transmission of SARS-CoV-2 [53].

Regarding a biological perspective, a more robust immune response to SARS-CoV-2 was demonstrated to be present in cats compared to dogs, and the risk of infection in pets is associated with the burden of human disease in their environment [54]. In addition, in America and Europe, cats and dogs respond differently to SARS-CoV-2 on their immune systems [31,55]. Compared to dogs, cats have demonstrated a greater seroprevalence and higher titers of neutralizing antibodies, suggesting a stronger immunological response [55]. Dogs, on the other hand, had a milder course of infection than cats because they showed differences in the results of the ELISA and serum neutralization test, which may have been caused by cross-reactions with other animal coronaviruses [56]. Research has indicated that cats are capable of shedding infectious viruses and directly infecting naïve cats, while experimental SARS-CoV-2 infection did not cause clinical illness in dogs [57].

The risk for SARS-CoV-2 seroprevalence in stray animals was significantly lower than in domestic animals [42]. In this context, it is clear that there is a need for a joint analysis of epizootiological and social risks in the transmission of SARS-CoV-2 to seroprevalence, considering that certain social and animal habits and ecological interactions in the environment cannot influence the greater transmission of the virus [58]. These can be divided into habits that contribute to or reduce transmissibility. Among the behaviors and contributory elements, the following can be mentioned: greater close contact between people and animals, lack of sufficient protective equipment and testing for populations, low purchasing power and quality of life, close contact between domestic and wild animals, illegal animal trafficking and the phenomenon of urbanization [59]. With regard to transmission restrictions for dogs and cats, the following can be exemplified: the use of appropriate means of transporting animals and a reduction in amateur tourism, wildlife and canoeing [60].

In Europe, there was disagreement about the data in this work, which may arise from different geographic, environmental, sociocultural and biological contexts. A study by Dróżdż et al. (2021) also demonstrates the presence of a very scattered and inconclusive picture regarding the higher prevalence of SARS-CoV-2 in these domestic animals investigated here in the European context, which may be related in some countries more to dogs (such as Bosnia and Spain), and in others to cats (such as France and Switzerland), while in others there is a high level of the two aforementioned animals (such as Italy, Holland and Germany) [58]. It was also observed in this present study that Asia and Africa have significant gaps in the literature data on SARS-CoV-2 infections in domestic animal species (including dogs and cats) and also in wild ones, indicating the need for monitoring and more rigorous investigation. This observation was also reported in a study by Fang et al. (2024) and is corroborated by this present study, which found no research related to infection in canines and felines on these continents [7].

This study is subject to limitations of possible information bias in the studies found for analysis, whether due to little multidisciplinary interaction of data in the available articles evaluated, the different method of detecting the virus in these animals in each study, defining a case of SARS-CoV-2 infection in each study and different ecosystem and fauna contexts in each region of the world or interactions of anthropozoonotic changes in the environment (including the pandemic) that can interfere with the seroprevalence of SARS-CoV-2 in dogs and cats [61].

This is a pioneering study on this topic in relation to measuring the potential risk of infection of these human companion animals. The presence of SARS-CoV-2 in animals as reservoirs can pose risks to the well-being and conservation of wildlife, in addition to hindering the control of the virus in humans [62]. Although the resulting mutant virus is not pathogenic to humans, it can be harmful to the respective animal species themselves and threaten the conservation of endangered species [63].

Therefore, more epidemiological, spatio-temporal, serological and experimental studies are needed to better assess the community ecological impact of the virus in the faunal biological context of these animals. Furthermore, regular epidemiological and genomic surveillance of animal hosts is recommended to detect and prevent new strains of the virus [63,64]. It is also recommended to vaccinate pets and captive animals to prevent herd events [65]. In the animal–human–environmental context, sustainable One Health surveillance is recommended to identify and prevent possible pandemics and epidemics caused by the disease [66].

A thorough examination of zoonotic viruses in different animal species and of workers most likely to be exposed to zoonotic hazards, such as farmers or butchers, would be necessary for early detection and to be prepared for the next possible epidemic [67]. Monitoring programs where the health of employees, animals and the general public are jointly monitored should be implemented by public health agencies, hygienists, occupational physicians, general practitioners and veterinarians [68]. The One Health concept must be included by governments in their public health systems, as well as in their education and training initiatives in implementing programs, policies and research to predict, prevent and clinically care for people [69].

In line with the limitations of this present meta-analysis, the use of the aforementioned keywords, the different collection period of animal samples for evaluation and the quality of evidence of the data in each article can be cited. However, an independent analysis was carried out to solve each confounding factor mentioned above; in relation to the selection of the best descriptors, a standardized evaluation system for all types of studies included simultaneously, and the sample collection periods for all studies were in times of COVID-19 outbreaks.

## 5. Conclusions

The COVID-19 pandemic context has made some scientific difficulties more noticeable in terms of understanding both the niche, biological, ecological, anthropological and environmental aspects of the causality of infections in animals and their relationship with humans. Among companion animals (dogs and cats) it was possible to verify in this meta-analysis that there is a high degree of risk of infection in cats compared to dogs. This was confirmed for analyses worldwide and on the American continent; however, no significant association was found on the European continent. Therefore, more epidemiological and experimental studies on the seroprevalence of SARS-CoV-2 in dogs and cats are necessary so that it is possible to create a generalized overview of the problem and in light of the most diverse factors and contexts applicable by region of the world.

## Figures and Tables

**Table 1:** Characteristics of the studies included in this systematic review.

No.	Reference	Database/Methodology	Quantity of Animals/Country of Origin	Results
1	Barua et al., 2021 [21]	PUBMED/Experimental study/ELISA test and sVNT	2092 animals/USA	6/956 cats (0.63%) and 5/1136 dogs (0.44%) tested positive for SARS-CoV-2.
2	Calvet et al., 2021 [22]	PUBMED/Experimental study/RT-PCR and PRNT	10 cats and 29 dogs/Brazil	Four cats (40%) and nine dogs (31%) from 10 study households were infected or seropositive for SARS-CoV-2.
3	Sánchez-Montes et al., 2022 [23]	PUBMED/Experimental study/qPCR test	30 cats and 100 dogs/Mexico	No detection in domestic animals (0%).
4	Villanueva-Saz et al., 2022 [24]	PUBMED/Experimental study/ELISA	114 cats/Spain	A total of four cats (3.51%) had a positive immunoenzymatic assay for detection of SARS-CoV-2
5	Michelitsch et al., 2020 [25]	PUBMED/Experimental study/ELISA and indirect immunofluorescence test	920 cats tested from Germany	Only six (0.69%) tested positive.
6	Michelitsch et al., 2021 [26]	PUBMED/Experimental study/ELISA and sVNT	1173 domestic cats/Germany	A total of 16 (1.36%) had circulating antibodies to SARS-CoV-2.
7	Klaus et al., 2021 [27]	PUBMED/Indirect ELISA and SARS-CoV-2 Surrogate Virus Neutralization Test (sVNT)	24 cats and 94 dogs/Germany and Italy	Only one dog (0.11%) and no cats (0%) tested positive for SARS-CoV-2.
8	Schulz et al., 2021 [28]	PUBMED/Experimental study/Virus neutralization test and ELISA	2160 cats/Germany, England, Italy and Spain	A total of 92 cats (9.3%) tested positive for the virus.
9	Goryoka et al., 2021 [29]	PUBMED/Experimental study/Virus neutralization assay test	56 animals/USA	4/19 cats (21%) and 4/37 dogs (10%) positive.
10	Hamer et al., 2021 [30]	PUBMED/Experimental study/Virus neutralization assay test	76 animals/USA	SARS-CoV-2 was detected in 7/16 cats (43.8%) and 7/59 dogs (11.9%) of the samples.
11	Patterson et al., 2020 [31]	PUBMED/Experimental study/Serological test	642 animals/Italy	A total of 11/191 cats (5.8%) and 15/451 dogs (3.3%) tested positive.
12	Fritz et al., 2021 [32]	PUBMED/Experimental study/Microsphere and immunoassay	47 domestic animals/France	A total of 8/34 cats (23.5%) and 2/13 dogs (15.04%) had samples confirmed for SARS-CoV-2.
13	Smith et al., 2023 [33]	PUBMED/Experimental study/Virus neutralization test	558 animals/England	A total of 2/186 cats (1.08%) and 4/372 dogs (1.08%) were positive.
14	Stevanovic et al., 2021 [34]	PUBMED/Experimental study/Microsphere and neutralization assay	787 animals/Croatia	1/131 cats (0.76%) and 2/656 dogs (0.31%) tested positive.
15	Zhao et al., 2021 [35]	PUBMED/Experimental study/Indirect ELISA and virus neutralization tests	1000 animals/Netherlands	A total of 2/500 cats (0.4%) and 1/500 dogs (0.2%) tested positive.
16	Yilmaz et al., 2021 [36]	PUBMED/Experimental study/ELISA and sVNT	155 cats/Turkey	A total of 34 cats tested positive (21.9%).
17	Pomorska-Mól et al., 2021 [37]	PUBMED/Experimental study/ELISA	622 animals/Poland	5/279 cats (1.79%) and 4/343 dogs (1.17%) had positive test results for SARS-CoV-2.
18	Cossaboom et al., 2021 [38]	PUBMED/Experimental study/rRT-PCR test	96 animals/USA	Of the 54 cats and 42 dogs, no animal (0%) tested positive for the presence of SARS-CoV-2-neutralizing antibodies.
19	Jaramillo Hernández et al., 2023 [39]	PUBMED/Experimental study/ELISA test	435 animals/Colombia	9/135 cats (6.67%) and 11/300 dogs (3.67%) had positive SARS-CoV-2 detection.
20	Ehrlich et al., 2023 [40]	PUBMED/Experimental study/rRT-PCR	275 cats/USA	None (0%) had antibodies to SARS-CoV-2.
21	Dias et al., 2021 [41]	PUBMED/Experimental study/rt-PCR test	96 animals/Brazil	A total of 1/49 cats (2.04%) and 1/47 dogs (2.13%) tested positive for the presence of SARS-CoV-2 RNA.
22	Barroso-Arévalo et al., 2023 [42]	PUBMED/Experimental study/ELISA and sVNT	1836 animals/Spain	27/817 cats (3.3%) and 38/1019 dogs (3.73%) tested positive for SARS-CoV-2.
23	Stanojevic et al., 2022 [43]	PUBMED/Experimental study/ELISA	105 animals/Serbia	2/36 cats (5.55%) and 1/69 (1.45%) dogs positive for SARS-CoV-2.
24	Bessière et al., 2022 [44]	PUBMED/Experimental study/ELISA and sVNT	308 animals (143 cats and 165 dogs)/France	12 cats (8.4%) and 9 dogs (5.5%) had neutralizing antibodies to SARS-CoV-2.

**Table 2:** Quality assessment of studies by ARRIVE guidelines.

Animal Class (Number of Studies)	Quality of Animal Studies as per ARRIVE Guidelines		
	Good (≥75%)	Moderate (≥65%)	Poor (≤55%)
Cats (14)	5 (35.71%)	9 (64.29%)	-
Dogs (10)	3 (30%)	7 (70%)	-
Total	8 (33.33%)	16 (66.67%)	-

## Data Availability

The original contributions presented in the study are included in the article. Further inquiries can be directed to the corresponding author.
